# Primordial odontogenic tumor: An immunohistochemical profile

**DOI:** 10.4317/medoral.21859

**Published:** 2017-04-08

**Authors:** Ronell Bologna-Molina, Toshinari Mikami, Vanesa Pereira-Prado, Fabio-Ramoa Pires, Roman Carlos-Bregni, Adalberto Mosqueda-Taylor

**Affiliations:** 1 Molecular Pathology Area, Faculty of Dentistry, Universidad de la República, Montevideo, Uruguay; 2 Associate Professor, Division of Anatomical and Cellular Pathology, Department of Pathology, Iwate Medical University, Iwate, Japan; 3 School of Dentistry, State University of Rio de Janeiro, Rio de Janeiro, Brazil; 4 Pathology Section, Head and Neck Clinical Center/Hospital Herrera-Llerandi, Guatemala City, Guatemala; 5 Health Care Department, Universidad Autónoma Metropolitana Xochimilco, Mexico

## Abstract

**Background:**

Primordial Odontogenic Tumor (POT) is a recently described odontogenic tumor characterized by a variably cellular loose fibrous tissue with areas similar to the dental papilla, covered by cuboidal to columnar epithelium that resembles the internal epithelium of the enamel organ, surrounded at least partly by a delicate fibrous capsule. The purpose of this study was to investigate the possible histogenesis and biological behavior of this rare tumor by means of a wide immunohistochemical analysis of its epithelial and mesenchymal components.

**Material and Methods:**

The immunoexpression of twenty-three different antibodies were evaluated in four cases of POT.

**Results:**

The epithelial cells that cover the periphery of the tumor showed immunopositivity for Cytokeratins 14 and 19, while Amelogenin, Glut-1, MOC-31, Caveolin-1. Galectin-3, PITX2, p53, Bax, Bcl-2, Survivin and PTEN were variably expressed in focal areas. The mesenchymal component of the tumor was positive for Vimentin, Syndecan-1, PITX2, Endoglin (CD105), CD 34, Cyclin D1, Bax, Bcl-2, Survivin and p53. PTEN and CD 90 showed a moderate positivity. BRAF V600E and Calretinin were negative in all samples. Cell proliferation markers (Ki-67, MCM-7) were expressed in <5% of the tumor cells.

**Conclusions:**

According to these immunohistochemical findings, we may conclude that POT is a benign odontogenic tumor in which there is both epithelial and mesenchymal activity during its histogenesis, as there is expression of certain components in particular zones in both tissues that suggests this tumor develops during the immature (primordial) stage of tooth development, leading to its inclusion within the group of benign mixed epithelial and mesenchymal odontogenic tumours in the current World Health Organization classification of these lesions.

** Key words:**Immunohistochemistry, jaw tumors, odontogenic, primordial.

## Introduction

Odontogenic tumors derive from the tooth-producing tissues or its remnants, which are entrapped either within the jawbones or in the adjacent soft tissues. Biologically, some of these lesions are hamartomas that show varying degrees of differentiation, while the others comprise both benign and malignant neoplasms exhibiting variable aggressiveness and potential for metastasis (1).

In 2014, our group reported for the first time an odontogenic tumor characterized by a variably cellular loose fibrous tissue with areas similar to the dental papilla, entirely surrounded by cuboidal to columnar epithelium that resembles the internal epithelium of the enamel organ and covered at least partly by a delicate fibrous capsule. The fact that the epithelium is located peripherally recalls the relationship between the immature inner enamel epithelium and the primitive dental papilla at a time when no inductive ecto-mesenchymal effects have occurred, and then it mimics the primordial stages of tooth development, apparently lacking the ability to follow a normal inductive evolution to produce the subsequent stages histo and morpho-differentiation of the dental tissues. This is the reason why we proposed the name “Primordial Odontogenic Tumor” (POT) as a descriptive term for this entity (2).

To date, seven cases of POT have been reported in the literature, all of which were found as well-defined radiolucent lesions adjacent to the crown of an unerupted tooth, producing varying degrees of bone expansion, root resorption and displacement of adjacent teeth, and macroscopically appeared as whitish solid tumors that tended to be encapsulated or at least well-defined from the surrounding structures (2,3).

The aim of this study was to better characterize POT both histopathologically and immunohistochemically, through the use of a large panel of antibodies. A further objective was to discuss the possible implications or functions held by each of the analyzed proteins in order to elucidate the possible histogenesis and biological behavior of this rare tumor.

## Material and Methods

Four cases of POT retrieved from three different Centers of Oral Pathology in Latin America (Two from Mexico, one from Brazil and one from Guatemala) were included in the present study. All of them belong to the series of six cases originally published by our group in 2014 (2). New immunohistochemical studies were performed with a panel of 23 different antibodies.

Paraffin blocks were cut and three-μm sections were set on glass slides previously treated with poly-lysine; we then proceeded to deparaffinize and rehydrate the slides and to perform antigen retrieval through treatment with 0.1 M sodium citrate (pH 6.2) and Tween 20 in microwave to unmask the epitopes. Endogenous peroxidases were blocked with 0.9% hydrogen peroxide. Primary antibodies for Amelogenin, CK19, CK 14,Vimentin, Calretinin, Syndecan-1, Glut-1, Galectin-3, Ki-67, MCM-7, Cyclin D1, PTEN, p-53, PITX2, Caveolin-1, BRAFV600E, Epithelial Related Antigen (MOC-31), CD-34, Endoglin (CD105), CD-90, Bax, Bcl-2 and Survivin were incubated for 45 min ( Table 1). Afterwards the slides were incubated with a biotinylated anti-mouse/anti-rabbit antibody and the streptavidin/peroxidase complex for 30 minutes each (LSAB þ-labeled streptavidin-biotin, Dako). To visualize the reaction a 3,30-diaminobenzidine-H2O (Dako) substrate was used. Finally, the sections were counterstained with Mayer’s hematoxylin solution. For the negative controls, the primary antibody was replaced with PBS.

**Table 1:**
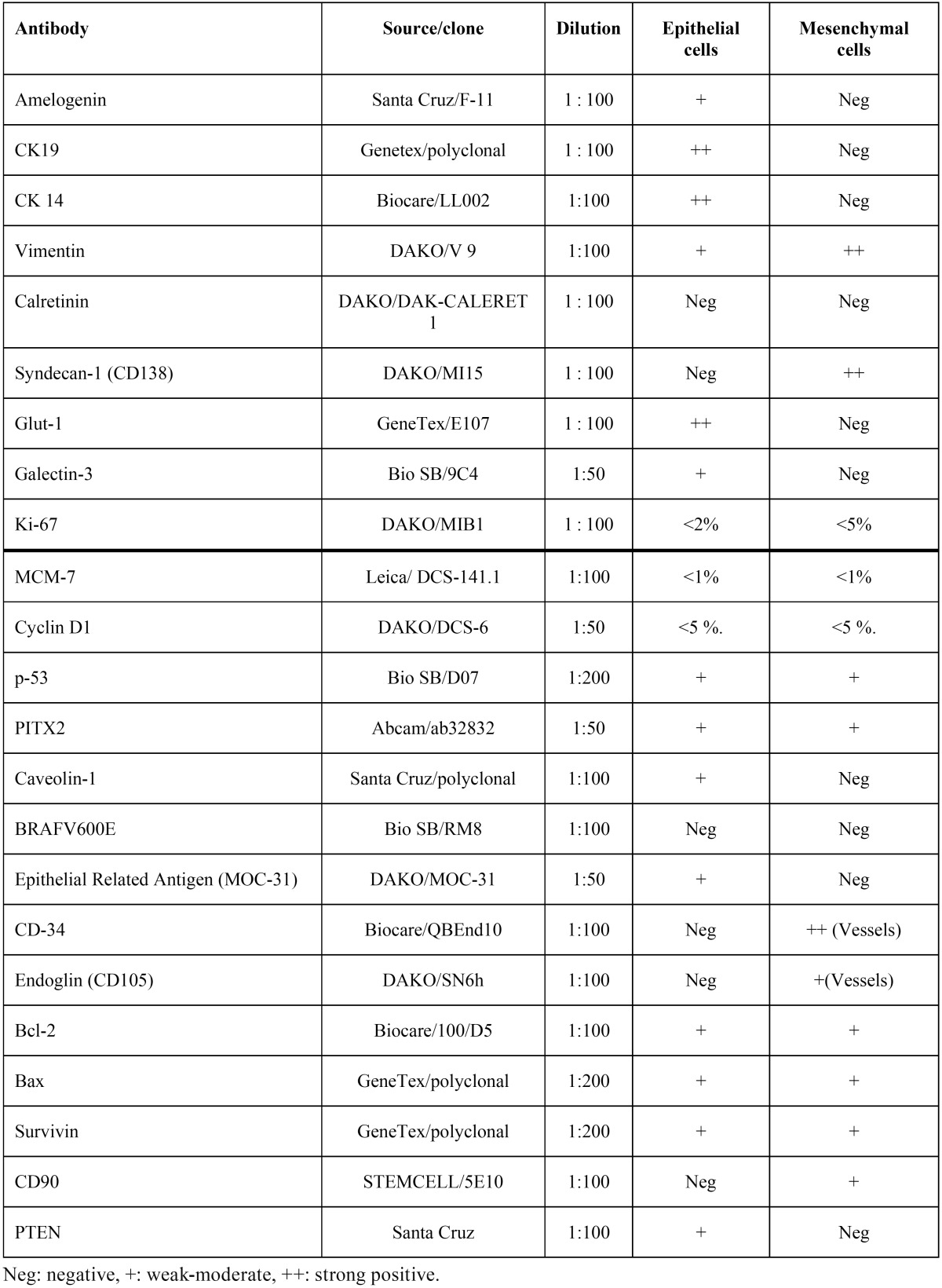
Immunoexpression of the 23 studied antigens in the epithelial and mesenchymal cells of POT samples.

For the cytoplasmic and/or membranous positivity the quantification was performed visually using an optical microscope (Eclipse Ci-L, Nikon, Japan) within 10 high-power fields/slide at the 40X objective amplification according to the following semi-quantitative scale. A score of 0 (“essentially no staining”) was established for negative or positive immunohistochemical staining of < 5% of the cells; + (“weak-moderate”) for staining of 5 to 50% of cells, and ++ (“strong positive”) for >50% positive staining. When the positivity was in the nucleus, the cell counts were performed as previously described by Bologna-Molina R, *et al.* (4). The labeling index (number of positive tumor cells/total number of tumor cells expressed as a percentage) was calculated in every case. The cell count was performed both in the mesenchymal component of the tumor and on the epithelial component surrounding each lesion. Two different oral pathologists assessed the immunohistochemical expression individually before reaching a consensus. The standardization of the examiners showed a kappa index of 0.90.

## Results

The clinical data of the four cases, including sex, age, location, clinical and radiographic findings, treatment and follow-up were described in our previous work (2) and are summarized in  Table 2.

**Table 2:**
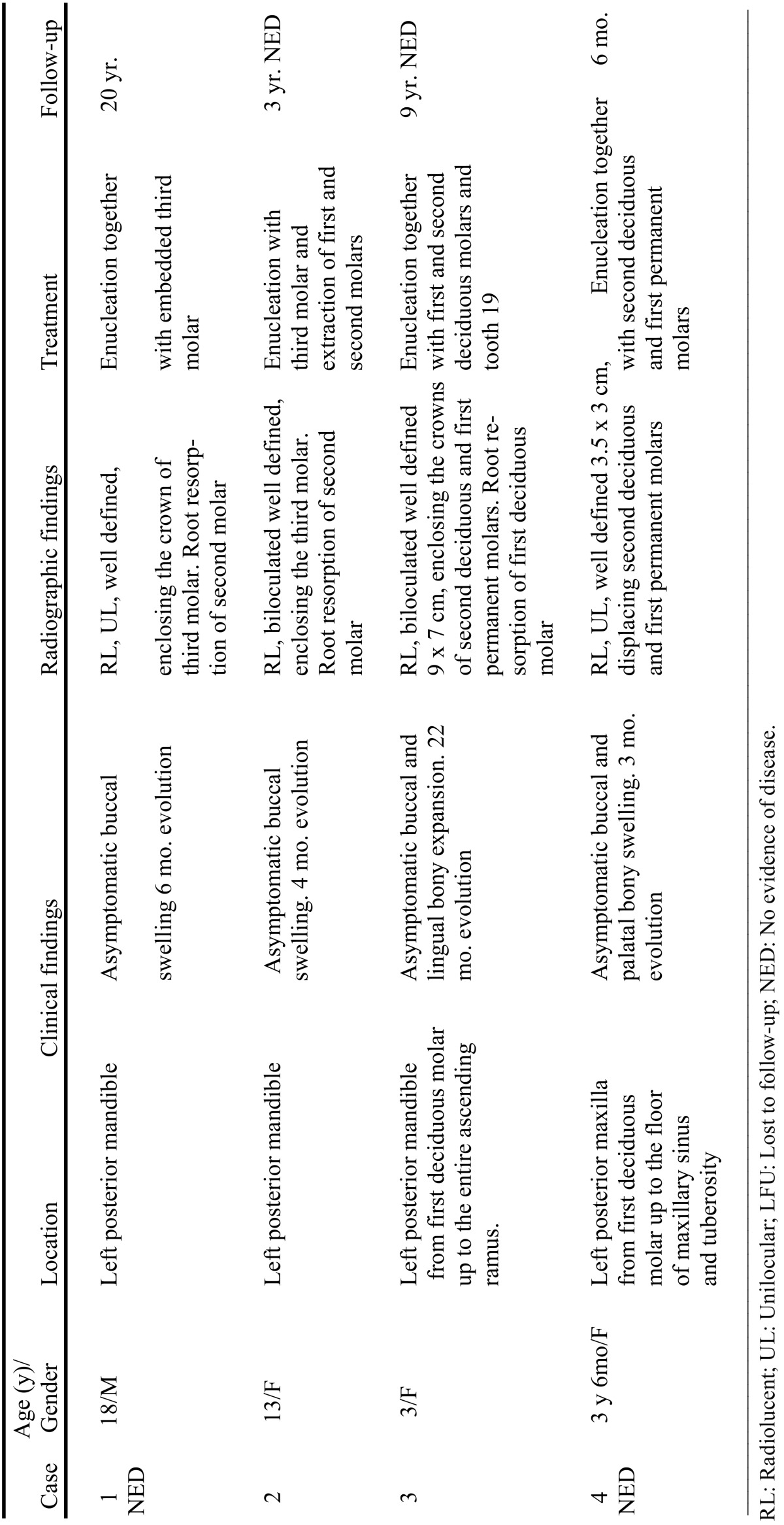
Clinico-pathological and radiographic features of 4 cases of Primordial Odontogenic Tumour.

**Figure 1:**
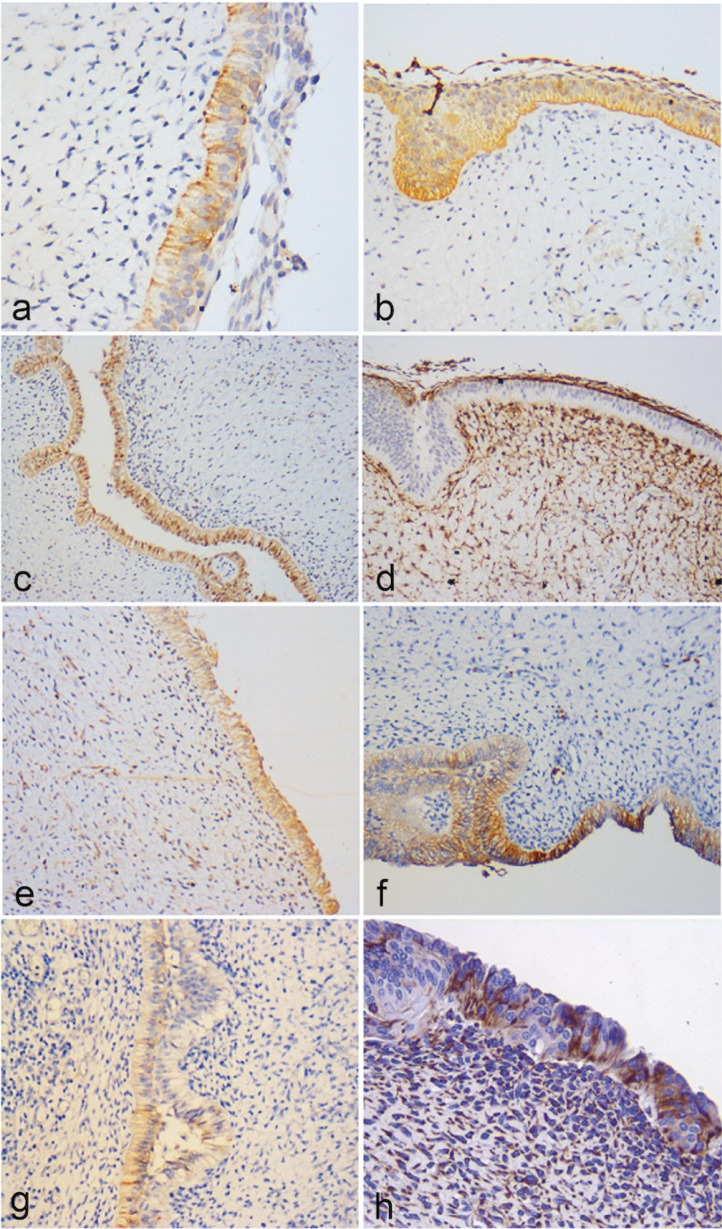
(a) The epithelium showS variable expression of CK19, 400X. (b) Expression of CK 14 was constant throughout the epithelium, 200X. (c) Expression of Amelogenin in the cubic and columnar cells, 200X. (d) Syndecan-1 is intensely expressed in the mesenchyme, but not in all the epithelial lining, 200X. (e) PITX2 positivity in focal areas, 400X. (f) Expression of GLUT-1 in the odontogenic epithelium, 200X. (g) MOC-31 was only found expressed in localized areas within the epithelium, 200X. (h) Some epithelial cells showed positivity for vimentin, 400X.

**Figure 2:**
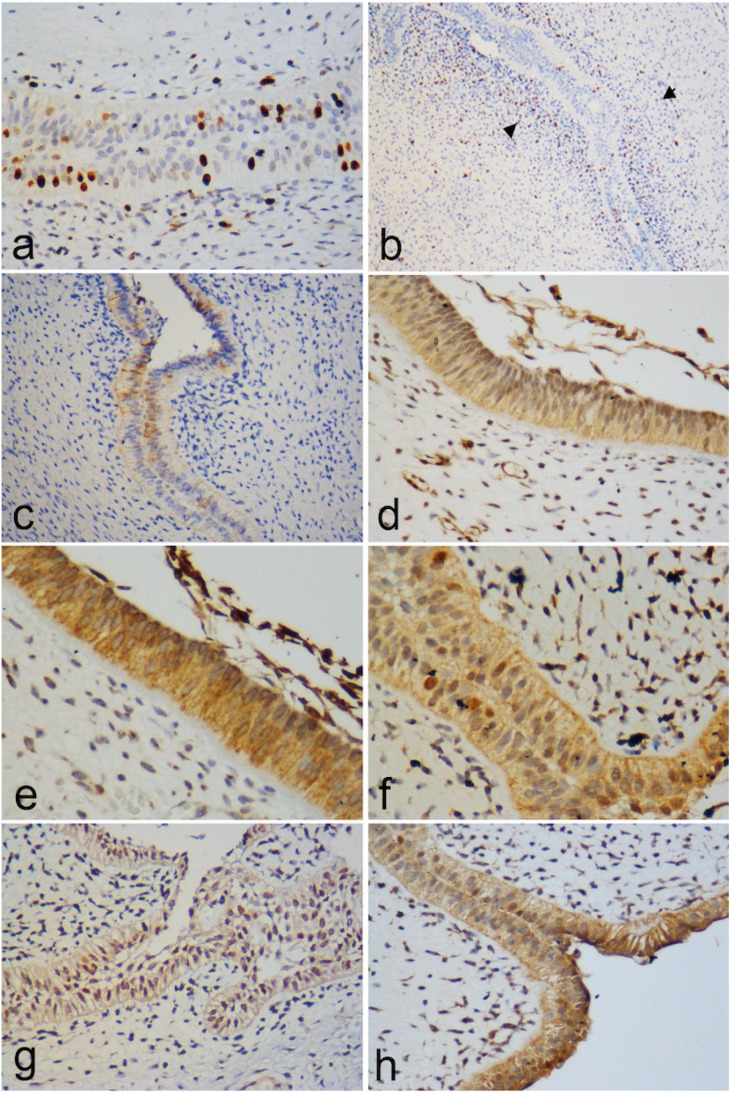
(a) Expression of Ki-67 in epithelial cells; 400x, (b) significantly higher proliferative index within the cells in the subepithelial mesenchymal condensation (arrow) as compared to that found in other mesenchymal areas of the tumor; 200X. (c) Expression of Galectin-3 in epithelial cells; 100X. (d,e,f) Bax, Bcl-2 and Survivin expression; 400X. (g) p53 was found both in the mesenchymal component and in the surrounding epithelium; 400X. (h) Cyclin D1 expression; 400X.

**Figure 3:**
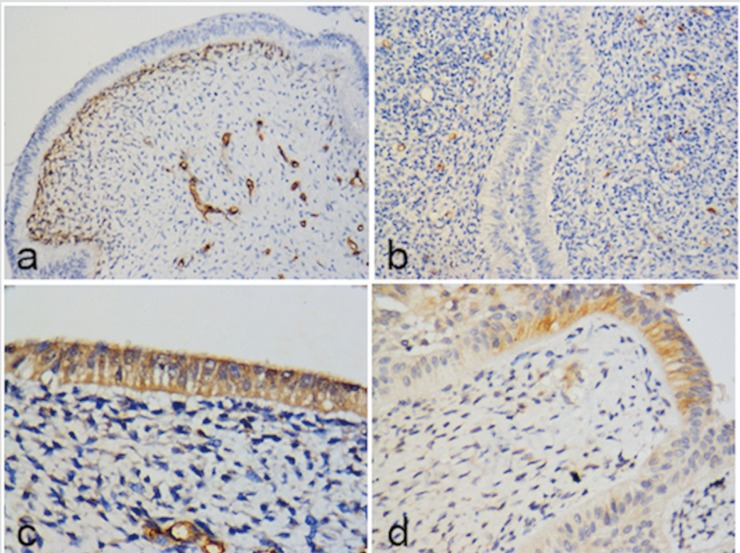
(a) Strong CD34 positivity in mesenchymal cells located in contact or closer to the odontogenic epithelium and in blood vessels; 200X. (b) CD 105 mainly expressed in blood vessels located in the areas of greater condensation of mesenchymal cells, particularly in the subepithelial region, thus signaling neoangiogenesis activity in this tumoral area; 200X. (c) Expression of CAV-1 in odontogenic epithelium; 400X (d) Slight expression of PTEN in focal areas of the epithelium; 400X.

The immunohistochemical findings show that the mesenchymal component of the tumor was positive in the fusocellular cells for Vimentin, Syndecan-1, PITX2, Bax, Bcl-2, Survivin and p53. Syndecan-1 positivity was also observed in the extracellular matrix. CD-34 and CD-105 (Endoglin) were variably positive in blood vessels, while PTEN and CD 90 showed weak-moderate positivity (see  Table 1 and Figs. 1-3). The epithelial cells that surround the periphery of the tumor were positive for CK 14 and in most areas also for CK19, Amelogenin, Glut-1, Epithelial Related Antigen (MOC-31) and Caveolin-1. Galectin-3, PITX2, p53, Bax, Bcl-2, Survivin and PTEN were variably expressed. On the other hand, BRAF V600E and Calretinin were negative in both the mesenchyme and the epithelium.

## Discussion

This study widely describes the immunohistochemical profile of POT. The selected markers provide new and valuable information for a better understanding of the possible histogenesis and biological behavior of this entity. Previous studies have demonstrated that immunoexpression patterns of CKs during odontogenesis vary with respect to specific cell types, as well as to the diverse developmental stages and the degree of differentiation of the epithelium (5). Although several CKs are expressed in the epithelial cells of the tooth germ (6), CK 14 is the one that seems to be present constantly in all stages of tooth development and in most neoplastic epithelial cells in odontogenic tumors (7-9), in contrast to CK 19, which increases its expression once the inner epithelial cells become potential ameloblasts (8). On the other hand, immunostaining of amelogenin in young or immature enamel matrix areas occurs in around 95% of the enamel protein. This protein, which is synthesized in young ameloblasts, is secreted into enamel matrix (10) and is gradually almost entirely removed by degradation by extracellular enzymes during enamel maturation (11). The presence of CK 19 and CK14 as well as the observed immunopositivity for Amelogenin in the epithelium of our cases of POT is a supporting evidence of an odontogenic origin of this tumor (Figs. 1a-c). It is interesting to note that both Amelogenin and CK 19 did not exhibit a uniform expression in all epithelial cells, as these predominated in the cubic and columnar cells showing inverse polarization (pre-ameloblasts), which suggests that this epithelial lining express diverse degrees of maturation, supporting an origin from primitive (primordial) cellular components of the enamel organ (Figs. 1a-c). This observation is also reinforced by the fact that some areas of the tumor epithelium showed focal positivity to vimentin (Fig. 1h).

MOC-31 is an epithelial-related antigen expressed in most normal epithelia but also in tumors originating from epithelial tissues. This is a cell adhesion molecule that allows tight junction formation between epithelial cells, which can negatively affect cadherin/catenin complex formation (12). MOC-31 has been proved to be an advantageous marker at signaling epithelial cell differentiation. Reduced expression of MOC-31 has been correlated with poor survival in cancerous conditions. It could be related to differentiation, tumor proliferation and invasion (13,14). This molecule was found only expressed in localized areas within the epithelium of the studied POT cases (Fig. 1g), suggesting, as seen for CK 19, Vimentin and Amelogenin, that there is a variable degree of differentiation and maturation of the cells that comprises the epithelial compartment.

As a member of the family of transmembrane heparan sulfate proteoglycans, Syndecan-1 (CD138) is involved in cell to cell adhesion and in the interaction of cells with the extracellular matrix, and also participates in promoting cell proliferation and regulates cellular growth by interacting with families of growth factors in association with heparin (15). Syndecan-1 plays a role in epithelial maturation, and it has been consistently observed that the degree of epithelial differentiation is directly associated with its expression (16). In addition, it has been reported that the pattern of Syndecan-1 expression varies according to the stage of maturation of the epithelial tissues (17). In this respect, it is relevant to highlight the fact that in all cases of POT included in this study, there were different degrees of Syndecan-1 expression in the tumoral epithelium, ranging from entirely negative areas to focally positive zones (Fig. 1d). This suggests that there are groups of epithelial cells with different degrees of maturation within the epithelial compartment of this tumor, which coincides with the differences in expression observed with MOC-31, CK19 and amelogenin. On the other hand, it was also noted a strong stromal expression of Syndecan-1 in areas of mesenchymal condensation (papilla-like tissue), particularly in the subepithelial regions (Fig. 1d). Vaino *et al.* reported that Syndecan RNA accumulates in the condensing mesenchymal cells around the invaginating epithelial tooth bud during early development, and this accumulation is more intense when morphogenesis advances towards the cap stage, and also that Syndecan expression is lost during the bell stage (18). The same group found that Syndecan-1 was intensely expressed in the mesenchyme, but not in the epithelium, by proliferating cells during the process of odontogenesis, suggesting a possible interaction of these tissues with growth factors (Fig. 1d) (19). These facts led us to consider that this lesion mimics the early (primordial) stages of tooth development.

PITX2 is selectively expressed in the early stages of morphogenesis in oral ectoderm and epithelial cells. Fine-tuned molecular signaling between the oral epithelium and the ectomesenchyme is involved in tooth development and it is considered a marker for the initiation of dental development (20,21). *In vitro* studies in human and mouse cells have shown that a mesenchymal signal is required to maintain PITX2 expression in the epithelium throughout tooth development (22). In the present study we found weak immunostaining for PITX2 in the fusiform mesenchymal cells in the four cases of POT, whereas the endothelial cells presented weak to moderate positivity (Fig. 1e). On the other hand, epithelial cells showed moderate positivity in focal areas (Fig. 1e). According to Mucchielli *et al.* (22), PITX2 expression occurs in the area of the presumptive dental lamina, remaining as a marker for dental epithelium throughout morphogenesis until it is downregulated in differentiated ameloblasts. The presence of this transcription factor, at least focally in the tumoral epithelium, also lend support to the hypothesis that this tumor derives from the early stages of dental morphogenesis and that the epithelial components express different stages of tooth development.

In mammalian cells, glucose is fundamental for metabolism. A family of transporters, called glucose transporters or Gluts, mediates its passage across cell membranes. Glucose Transporter Glut-1 is found in normal cells, such as erythrocytes and endothelium at the blood-brain barrier in perineural cells, and in the basal layer of oral epithelium and epidermis. Conditions in which the metabolic rate must be adjusted can induce increased glucose transport via Glut-1. Examples of such conditions are cell division, differentiation, nutrient starvation and hypoxia, which are frequently found in embryological tissues and in those with malignant transformation (23). Glut-1 is also expressed in odontogenic tumors, and it has been observed increased in ameloblastomas and ameloblastic carcinomas as compared to tooth germs, which suggests its overexpression may play an important role in the aggressive behavior of these neoplasms (24). In the present study we found a strong expression of this glucose transporter in odontogenic epithelium, showing both cytoplasmic and membranal localization (Fig. 1f), which correlates well with the fact that the pattern of Glut-1 expression changes from strong cytoplasmic expression to membranous expression during maturation of the enamel organ in the late bell stage (24). The strong expression of this protein in the epithelium of POT indicates the existence of high glucose transport requirements in this pathological condition. On the other hand, the cytoplasmic and membranous expression also suggests the co-existence of different stages of maturation in this epithelium.

In order to quantify the proliferative activity of the tumor we used markers associated to cell proliferation and factors related to cell –cycles, such as Ki-67, MCM7 and Cyclin D1, which revealed that the tumor presents a low rate of proliferation (< 5%) in both epithelial and mesenchymal cells (Fig. 2a,b), similar to that reported for other benign odontogenic tumors, such as the odontogenic myxoma (25). Additionally, it is important to highlight the fact that there was a significantly higher proliferative index among the cells located within the subepithelial mesenchymal condensation as compared to that found in other mesenchymal areas of the tumor (Fig. 2b). This may imply that the tumor shows more active growth in this specific area, and perhaps it may be related to the inductive ability of that part of the mesenchyme to modify the overlying epithelium.

Galectin-3, a lectin involved in diverse biological events including embryogenesis, cell adhesion, proliferation, apoptosis, mRNA splicing and regulation of the immune system (26), is widely present in human tissues. Changes in Galectin-3 expression and its subcellular and intercellular localization are commonly observed in cancer and precancerous conditions (27). While a general shift of galectin-3 localization from nucleus to cytoplasm occurs in cancer development from adenoma to carcinoma and also this protein plays an important pivotal role within the nucleus in the regulation of cancer-related gene expression (27), including Cyclin D1, its expression in POT in this study was exclusively found in the epithelial component, mainly in the cytoplasm (yet some cells showed immunopositivity in the nucleus) (Figs. 2c,h), suggesting a possible role in cell proliferation. As occurred with other immunomarkers, there were areas in the epithelium that were completely negative and some which showed clear expression (Fig. 2c). Nakahara *et al.* (28) have suggested that overexpression of intracellular galectin-3 favors neoplastic transformation, cell cycle progression and inhibition of apoptosis, but the exact significance of our findings remains to be elucidated.

The range of survival or cellular death is determined by the relationship between Bcl-2 (an apoptosis inhibitor) and Bax (an apoptosis promoter). In addition, Survivin is an antiapoptotic protein that leads to negative regulation of apoptosis by inhibiting caspase activation (29). When Bax predominates over Bcl-2, the activity of Bcl-2 is repressed and the apoptotic pathway of Bax is activated (30). In this study Bcl-2, Survivin and Bax manifested strong positivity in both mesenchymal and epithelial cells of POT (Figs. 2d,e,f). The expression of Bax, Survivin and Bcl-2 was similar, with slightly greater presence of the antiapoptotic proteins; however, the results of this study does not allow us to conclude that the overexpression of antiapoptotic proteins play an important role as growth mechanism of POT, as it has been suggested for other odontogenic neoplasms (31). It is interesting to note that nuclear immunoexpression of Survivin was detected in some areas of the epithelium (Fig. 2f), which according to Stasikowska-Kanicka *et al.* (32), fosters cell proliferation, while its cytoplasmic immunoexpression is involved in the mechanisms of apoptosis regulation. On the other hand, both Bcl-2 and Bax participate as transcriptional targets for p53, a tumor suppressor protein, which brings about cell cycle arrest or apoptosis in response to DNA damage. The coordinate activity of these molecules is vital in order to control life and death of a cell (33). A weak to moderate expression of p53 was found both in the mesenchymal component and in the surrounding epithelium in our cases (Fig. 2g), which is evidence that this protein participates as part of the regulation of this tissue.

Blood supply is required for tumor growth, to provide the oxygen, metabolites and growth factors necessary for cell proliferation. Blood vessels feeding the tumor were identified by means of CD34 and Endoglin (CD-105) expression, demonstrating strong positivity for CD34 in all the endothelial cells, whereas CD 105 marked a smaller number of blood vessels (Figs. 3a,b). This situa-tion may be explained by the fact that CD-105 expression, contrary to CD34, is a characteristic property of newly formed blood vessels and not of previously-existing blood and lymphatic vessels (34). CD 105 in POT was found mainly in blood vessels located in the areas of greater condensation of mesenchymal cells, particularly in the subepithelial region, thus signaling neoangio-genesis activity in this tumoral area (Fig. 3b), which agrees with our previous suggestion that the tumor has a greater growth rate in this area. In this recently recognized tumoral entity we found that CD34 showed immunopositive areas not only in the mesen-chymal vessels, but also in the mesenchymal cells located in contact or closer to the odontogenic epithelium (Fig. 3a). CD34 is known to be a marker for hematopoietic stem cells, and hematopoietic progenitor cells (35), but also it is regarded as a marker of several other nonhematopoietic cell types, including vascular endothelial progenitors and embryonic fibroblasts (36,37), and therefore we consider that its positivity in this area could indicate the presence of embryonic fibroblasts (38,39).

CD90 has been established as a marker for a wide variety of human cell-stem types, and particularly mesenchymal stem cells. It is considered to be a vital regulator, critical at cell-cell and cell-matrix interactions. It takes part in cellular migration and fibrosis, both of which are important events for tissue regeneration and oncogenesis (40). Its expression in tumor stroma, especially in endothelial and mesenchymal stem cells, including tumor-associated fibroblasts, appears to be an influential agent in disease progression (41). Stromal expression of CD90 in our POT cases was found in blood vessels and in some mesenchymal cells, particularly in those in close proximity to epithelial odontogenic cells, which led us to suggest that CD90 expression in POT could play an important role in cellular adhesion and migration, serving as an important regulator of cell-matrix interactions for determining the biological behavior of this tumor.

Expression of Caveolin-1 (Cav-1) was reported by Schwab *et al.* (42) in epithelial and mesenchymal cells of tooth germ in various stages of development, particularly in the inner enamel epithelium and ameloblastic cells in developing tooth germ (42), and it has also been established that Cav-1 participates in oncogenic transformation and differentiation in oral squamous cell carcinoma and prostate cancer (43,44). Regarding its tumor promoting function, higher expression of Cav-1 drives into tumorgenesis by inhibiting apoptosis (44). The variable expression that we found within the epithelial component of POT (Fig. 3c) could suggest different stages of cellular differentiation within the odontogenic epithelium covering the lesion, implying that positive cells are the transformed cells of this particular type of tumor.

PTEN gene, which in humans encodes the protein phosphatase and tensin homolog, (PTEN), was identified as a tumor suppressor gene that mutates with a high frequency in a large number of cancerous condition. Reduced expression of PTEN has also been reported in some oral cancers, in studies that suggest PTEN plays an important role at signaling pathways in the carcinogenesis of head and neck squamous cell carcinoma (45). In our study, we observed only a slight expression of PTEN in focal areas of the epithelium (Fig. 3d), reinforcing the hypothesis of the existence of cells with different stages of development within the epithelial compartment.

Recently, dysregulation of the MAPK pathway signaling have been proved as a critical step in the pathogenesis of ameloblastoma. The mutation most frequently identified is BRAF V600E. (46). To date, BRAF V600E mutations have been identified also in ameloblastic fibromas (2/2) and fibrodentinomas (1/1), as well as in ameloblastic carcinomas (3/8) and clear cell odontogenic carcinomas (1/1), but not in other odontogenic tumors (47,48). In our POT cases, we did not find the presence of the mutant protein BRAFV600E, and the absence of this mutation positions POT in a different category with respect to the ameloblastic lesions.

According to the variable extent of expression of the diverse proteins tested in this study, we may conclude that POT has a low proliferative index, which defines it as a slowly growing lesion with variable production of loose fibrous tissue in which cell proliferation does not seem to be the only growth mechanism implicated in its expansion. POT is a moderately vascularized tumor, in which there is greater vascular density and vascular neoformation in the mesenchyme located just below the epithelium, an area that also shows greater expression of Syndecan 1, CD 34, Ki-67 and Glut-1, indicating that this is the area with the greatest proliferative activity and in which the necessary elements for the development of the tumor converge. In addition, there is some evi-dence of an increase of antiapoptotic activity that may participate in the growth of this tumoral entity. The uneven expression of CK19, Vimentin, Amelogenin, Syndecan-1, CAV 1, Glut-1 1, MOC-31 and PITX2 in the surrounding epithelium could be inter-preted as that there are groups of cells in different stages of development within an apparently unique epithelial structure. This situation leads us to speculate that the epithelial compartment of POT is not a uniform or static structure in odontogenesis, but that it expresses the transition between the earliest stages of tooth development and those in which ameloblastic maturation is present, but before having the potential for induction of odontoblastic differentiation or the production of mineralized tissues, supporting the denomination of “primordial” for this unique entity. The absence of identifiable BRAF V600E mutant protein suggest that mutation of BRAF gene is not implicated in the pathogenesis of POT, and may confirm that this tumor does not belong to the category of those BRAF-mutant positive ameloblastic tumors.

Considering and putting together all these immunohistochemical findings, we interpret that the lesion studied is an odontogenic tumor of benign behavior that possibly originates from the mesenchyme of an abortive tooth germ that fails to produce a dental organ. Given the rarity of this tumor and the small number of cases reported to date, it is necessary to collect new cases in order to obtain a larger sample, towards a better understanding of the histogenesis and tumoral behavior of this condition.
